# Hypofractionation: less is more?

**DOI:** 10.18632/oncotarget.28023

**Published:** 2021-08-17

**Authors:** Neethu Billy Graham Mariam, Yee Pei Song, Nuradh Joseph, Peter Hoskin, Kimberley Reeves, Nuria Porta, Nicholas James, Ananya Choudhury

**Affiliations:** ^1^Department of Clinical Oncology, The Christie NHS Foundation Trust, Manchester, UK; ^2^Division of Cancer Sciences, School of Medical Sciences, Faculty of Biology, Medicine and Health, University of Manchester, Manchester, UK; ^3^Ministry of Health, Colombo, Sri Lanka; ^4^Mount Vernon Cancer Centre, Northwood, UK; ^5^Clinical Trials and Statistics Unit, The Institute of Cancer Research, London, UK; ^6^Prostate and Bladder Cancer Research Team, The Institute of Cancer Research, London, UK

**Keywords:** hypofractionation, BC2001, BCON, bladder cancer

## Abstract

One third of patients with bladder cancer present with muscle invasive bladder cancer (MIBC) which has a poor prognosis. International guidelines for the management of MIBC recommend radical cystectomy or bladder-preserving treatment based on radical radiotherapy with a form of radiosensitisation. In the UK, both conventional fractionation with 64 Gy in 32 fractions and hypofractionation with 55 Gy in 20 fractions are standard of care options with the choice varying between centres. A meta-analysis of individual patients with locally advanced bladder cancer from two UK multicentre phase 3 trials was published recently. This study evaluated the non-inferiority of a hypofractionated schedule compared to a conventional regime. This analysis confirmed the non-inferiority of the hypofractionated regimen, and noted superior locoregional control. We discuss the relevance of these findings to current practice while considering the radiobiology of hypofractionation, the role of systemic therapies and radiosensitisation, as well as the socioeconomic benefits.

## INTRODUCTION

A meta-analysis of individual patients with locally advanced bladder cancer, from two multicentre phase 3 trials done in the UK, BC2001 and BCON, was published recently [1]. BC2001 assessed the addition of chemotherapy to radiotherapy and BCON assessed hypoxia-modifying agents combined with radiotherapy [2–4]. The study evaluated the non-inferiority of a hypofractionated schedule of 55 Gy in 20 fractions when compared to a conventional regime of 64 Gy in 32 fractions. Not only did the study confirm the non-inferiority of the hypofractionated regimen, superior locoregional control was noted with the shorter schedule. The two regimes had comparable rates of overall survival and toxicity.

### Why is this important and what does it mean for current practice?

Bladder cancer is estimated to account for 3% of cancer diagnoses worldwide and 3% of cancer-related deaths within the UK [5, 6]. One third of patients present with muscle invasive bladder cancer (MIBC) which has a poorer prognosis. International guidelines for the management of MIBC recommend radical cystectomy or bladder-preserving treatment based on radical radiotherapy with a form of radiosensitisation [7–9]. Most bladder-preserving schedules involve prior transurethral resection of bladder tumour (TURBT). There is level 3 evidence supporting the use of radiosensitisation with either chemotherapy or hypoxia modification [2–4, 10]. Neoadjuvant chemotherapy or immunotherapy may also precede radical cystectomy or radiotherapy [11–13].

Previous attempts to compare outcomes of cystectomy and radical radiotherapy in a randomised controlled trial closed prematurely due to difficulties in recruitment [14]. Nevertheless, large retrospective studies have demonstrated comparable rates of overall survival in patients undergoing the two treatments when known prognostic factors are accounted for [15]. In the UK, both conventional fractionation with 64 Gy in 32 fractions and hypofractionation with 55 Gy in 20 fractions are standard of care options with the choice varying between regional centres. The superiority of the hypofractionated regime in terms of invasive locoregional recurrence and comparable toxicity profiles, as shown in this metanalysis [1], strongly suggests that this regimen should be adopted as the standard of care for bladder preservation in patients with locally advanced bladder cancer. In the BC2001 trial 77.5% of patients receiving 20 fractions had invasive locoregional control by 3 years, relative to 74.2% for those receiving 32 fractions. In the BCON trial 63.5% of patients receiving 20 fractions had invasive locoregional control at 3 years relative to 56.2% for patients receiving 32 fractions. Local control was achieved at high rates in both trials despite high proportions of patients having incomplete resections. This suggests that TURBT may not be an essential step in bladder preservation and incomplete resection should not be an exclusion criterion. Incomplete TURBT is probably best viewed as merely a surrogate for a higher stage and therefore will also predict a poorer outcome with surgery or bladder preservation.

### What is hypofractionation and why does it work?

A fundamental concept of radiobiology is that a single dose of ionising radiation will be more effective at killing a cancer cell compared to the same dose divided into smaller fractions [16]. Fractionation is vital to protect normal tissues. The radiosensitivity of tissue is underpinned by an interplay between the 5 Rs of radiobiology: repair, redistribution, reoxygenation, repopulation and intrinsic radiosensitivity. The linear quadratic model of tissue radiobiological characteristics estimates that different tissues have dissimilar sensitivities to fractionation [17]. The α/β ratio quantifies fractionation sensitivity; α represents unrepairable lesions after radiotherapy, and is in effect a parameter of radiosensitivity, and β represents repairable sublethal lesions. Bladder cancer, which is considered a rapidly proliferating tumour, may be assumed to have a high α/β ratio and lower fractionation sensitivity. This means that convention has assumed that bladder cancer radiotherapy is best given as 2 Gy per fraction. However, the results of the meta-analysis of BCON and BC2001 with the superiority of moderate hypofractionation contradicts this convention. There are a number of potential explanations for this, but it is likely that accelerated repopulation is more important than previously thought and that the α/β ratio of bladder cancer is lower. In this case, the overall treatment time becomes crucial in improving the outcome of treatment. Mathematical modelling has suggested that accelerated repopulation kicks in after 5 weeks for bladder cancer meaning that treatment is best completed in less than 5 weeks [18].

A key concern with hypofractionation regimes is late toxicity. Long term patient-reported outcome measures (PROMS) for BC2001 show that there is no adverse impact from hypofractionation nor from the addition of chemotherapy to radiotherapy, with around two thirds of patients showing a long-term gain in PROMS compared to baseline [19]. Additionally, the BCON trial outcomes show no differences in late toxicities between treatment groups or fractionation schedules. Although there was no detriment in late toxicity for the hypofractionation schedule in the meta-analysis, with the advent of advanced radiation techniques in image guidance as well as intensity modulated radiotherapy, we are now able to better conform high-dose radiation to the target volume. This has the potential to reduce long-term side-effects further.

### The role of systemic therapies

Neoadjuvant chemotherapy has clinical benefit and is offered as standard of care in the UK prior to bladder preservation [8, 11, 12]. However, trials of neoadjuvant chemotherapy in bladder preservation predated the use of radiosensitising agents. Recent data from the BC2001 trial showed that radiosensitising chemotherapy improves locoregional control even in patients treated with neoadjuvant chemotherapy [20]. However, there is no robust evidence of benefit with the use of neoadjuvant chemotherapy in patients treated with radical radiotherapy and a radiosensitiser. The role of adjuvant chemotherapy is not as well characterised due to early closure and/or poor recruitment to trials resulting in a lack of level 1a evidence [21, 22]. There is much interest in immunotherapeutics in this group of patients, but for now, neoadjuvant, concurrent or adjuvant immunotherapy is for trial use only.

### What about radiosensitisation?

The combination of radiotherapy and a radiosensitising agent is thought to work synergistically to improve the overall clinical benefit achieved with radiotherapy alone [23]. A number of different radiosensitisers are used alone or in combination including 5-Fluorouracil (5-FU) with mitomycin-C (MMC), gemcitabine, cisplatin, carbogen and nicotinamide [7]. In BC2001, patients received either radical radiotherapy alone or with concomitant 5-FU and MMC whilst BCON compared hypoxia modification with carbogen and nicotinamide [2–4]. Of note, improvement in invasive locoregional control was seen with radiosensitisation in both trials. A hypoxic tumour microenvironment promotes tumour survival and progression through various mechanisms including induction of the angiogenic switch and alterations in cellular metabolism [24, 25]. Hypoxia also underpins treatment resistance to both radiotherapy and systemic agents. Hypoxia modifying agents, such as nicotinamide (vitamin B3 analogue) and carbogen (a mixture of 95–98% oxygen and 2–5% carbon dioxide), improve oxygenation of the tumour microenvironment hence mitigating this effect [26]. Despite decades of radiobiological studies in hypoxia modification, there has been poor uptake as standard of care [27]. The BCON biobank has been of great use in evaluating the potential of predictive biomarkers for hypoxia modification. Necrosis and a 24 gene transcriptomic signature can predict patients who respond to carbogen and nicotinamide [28].

### Socioeconomic benefits of hypofractionation

The rapid evolution of radiotherapy technology has been accompanied by mounting costs. Efforts to quantify and rationalise these costs by various groups, including the European Society for Radiotherapy and Oncology in the Quantification of Radiation Therapy Infrastructure and Staffing Needs study, have demonstrated the high degree of variability in radiotherapy resources including highly-skilled personnel across Europe [29, 30]. The Health Economics in Radiation Oncology project was launched to both give a complete picture of these disparities and to provide real-world solutions [31]. Although no such projects have been undertaken to assess the situation in low and middle-income countries (LMIC), The Global Cancer Observatory and International Atomic Energy Agency Directory of Radiotherapy Centres data has shed some light on the circumstances [32]. These data suggest that 50–90% of cancer patients in LMICs have limited access to treatment, with significant shortages in equipment and personnel [33]. There are huge potential gains to be made through the use of hypofractionated regimes in these settings; shorter regimes free up equipment, save patient and clinician time and have lower day-to-day costs such as transportation fees [34, 35]. Such gains, of course, also apply to high income countries.

The ongoing COVID-19 pandemic has had significant impacts on radiotherapy delivery, as detailed in a recent population-based study [36]. Though there was an overall fall in mean weekly radiotherapy courses during the first wave in the UK’s National Health Service, the number of courses of bladder radiotherapy increased by 64%. This may reflect increased use of radiotherapy as a preferred definitive treatment option during times of critical clinical pressures, faced particularly in cancer surgical services. Rapid guidelines issued by the National Institute of Clinical Excellence and the Royal College of Radiologists highlighted bladder cancer as priority level 1 (the highest priority level on a scale of 1–5) for the provision of radiotherapy [37, 38]. The adoption of ultrahypofractionated regimes, based on the FAST-Forward trial in the adjuvant setting for breast cancer [39, 40] and PACE-B or HYPO-RT for radical prostate cancer [41, 42], increased during the pandemic. There is little evidence for ultrahypofractionation in the radical treatment of bladder cancer, however, there was an increased uptake of hypofractionated regimes [36]. As a result, treatment attendances fell considerably even after lockdown easing due to the continued use of hypofractionated regimes [36], with the intended benefit of reducing footfall. It appears that the practicalities of hypofractionation, paired with optimal clinical outcomes, in times of great clinical pressures have led to its wider acceptance in a number of solid tumours including bladder cancer.

### Final word

The BC2001 and BCON meta-analysis supports the adoption of a hypofractionated schedule of 55 Gy in 20 fractions as standard of care in locally advanced bladder cancer due to superiority in locoregional control with comparable overall survival and no increase in side-effects. The 55 Gy in 20 fraction schedule can be safely delivered with radiosensitisation and there is level 1 evidence that this improves outcomes compared to radiotherapy alone. There are clear health economic benefits when adopting hypofractionated regimes with radio-sensitisation, with particular importance in future pandemic preparedness. These benefits may allow for more equitable distribution of resources, especially where there are limitations, and in times of severe clinical pressure.
